# Serum levels of S100A6 are unaltered in patients with resectable cholangiocarcinoma

**DOI:** 10.1186/s40169-016-0120-7

**Published:** 2016-09-27

**Authors:** Sven H. Loosen, Fabian Benz, Jennifer Niedeggen, Maximilian Schmeding, Florian Schüller, Alexander Koch, Mihael Vucur, Frank Tacke, Christian Trautwein, Christoph Roderburg, Ulf P. Neumann, Tom Luedde

**Affiliations:** 1 Department of Medicine III, University Hospital RWTH Aachen, Pauwelsstrasse 30, 52074 Aachen, Germany; 2 Department of Surgery, University Hospital RWTH Aachen, Pauwelsstrasse 30, 52074 Aachen, Germany; 3 Division of Gastroenterology, Hepatology and Hepatobiliary Oncology, University Hospital RWTH Aachen, Pauwelsstrasse 30, 52074 Aachen, Germany

**Keywords:** S100A6, Cholangiocarcinoma (CCA), Cancer, Biomarker, Prognosis, CA19-9, CEA

## Abstract

**Background:**

Elevated expression levels of S100A6, a calcium-binding low-molecular-weight protein, were demonstrated in various malignancies. Moreover, increased serum levels of S100A6 were suggested as a novel biomarker for various inflammatory and malignant diseases including lung and gastric cancer. However, up to now, serum concentrations of S100A6 have not been analyzed in patients with cholangiocarcinoma (CCA).

**Methods:**

S100A6 mRNA expression levels were analyzed in human and murine CCA tumor samples, using semi-quantitative reverse transcriptase PCR. S100A6 serum concentrations were measured using an enzyme-linked immunosorbent assay in 112 patients with CCA referred to surgery for curative resection and were compared to those of 42 healthy controls. Results were correlated with clinical data.

**Results:**

S100A6 mRNA expression levels were significantly up-regulated in tumor samples of CCA patients and in tumor tissue of a CCA mouse model. In contrast, serum levels of S100A6 were not significantly altered in patients with CCA compared to healthy controls. Whereas no differences became apparent within the different clinical subgroups of CCA, patients with primary sclerosing cholangitis (PSC)-based CCA displayed higher levels of S100A6 compared to the other patients. Nevertheless, patients with higher S100A6 serum concentrations showed a trend towards an impaired prognosis compared to patients with lower levels. Finally, within our cohort of patients both the diagnostic and prognostic potentials of S100A6 were similar to those of the clinically established biomarkers CEA and CA19-9.

**Conclusion:**

Although S100A6 was expressed at significantly higher levels in human and murine CCA tumor samples, S100A6 serum levels were not regulated in patients with CCA and are thus not suitable for diagnosis of CCA. However, CCA-patients with elevated S100A6 displayed a trend toward an impaired prognosis compared to patients with lower S100A6 levels, supporting its further evaluation as a prognostic biomarker in CCA.

**Electronic supplementary material:**

The online version of this article (doi:10.1186/s40169-016-0120-7) contains supplementary material, which is available to authorized users.

## Background

Cholangiocarcinoma (CCA) accounts for 10–15 % of all hepatobiliary malignancies and is therefore the second most common primary tumor of the liver [1]. According to its location, CCA can be classified as intrahepatic, perihilar or extrahepatic CCA [2]. The global incidence of CCA shows large geographical variations with incidence rates of 0.5–3.4/100,000 in Western Europe and the United States and up to 85/100,000 in Northeast Thailand [3]. Although the incidence of extrahepatic CCA seems to decrease slightly over time, the overall incidence of CCA and especially the incidence of intrahepatic CCA have shown a strong increase over the last decades [4–6]. Surgical resection remains the only potentially curative treatment option for all types of cholangiocarcinoma, but is often not feasible due to an advanced disease stage at diagnosis [2]. The standard therapy for patients with inoperable advanced stage CCA is a palliative chemotherapy with the substances gemcitabine and cisplatin [7–9].

The overall survival of CCA patients has remained fairly poor with a post-operative 5-year survival rate of 23–42 % after R0 resection and 0 % after R+ resection for intrahepatic CCA [10, 11] and 27–37 % for extrahepatic CCA, respectively [12, 13]. For advanced tumor stages, the 5-year survival rate has remained below 5 % [14, 15], highlighting the urgent need for biomarkers allowing an early diagnosis and prognosis of the disease. As cholangiocarcinoma can be considered one of the less common types of cancer, there are only a limited number of studies evaluating potential biomarkers for CCA. To date, no serum based marker detecting CCA with an appropriate sensitivity and specificity at early stages of disease could be established.

The S100 protein family consists of more than 25 low-molecular-weight proteins, characterized by Ca^2+^-binding EF-hand motifs [16]. S100A6 (calcyclin) is a 90-amino-acid, 10.5 kDa protein that is predominantly expressed in the cytosol of numerous human cells, including fibroblast, epithelial cells, neuronal cells, lymphocytes, platelets, cardiomyocytes and smooth muscle cells [17–23]. A large number of studies have associated S100A6 with the development of cancer [17]. As such, S100A6 was shown to be significantly up-regulated in the tumor tissue of cutaneous melanoma, colorectal adenocarcinoma, stomach and thyroid cancer, astrocytoma or pancreas ductal adenocarcinomas [24–29]. Functionally, S100A6 was shown to play a decisive role in different molecular processes in tumorigenesis. In renal cell carcinoma cells, knockdown of calcium-regulating S100A6 suppressed cell growth via induction of G2/M phase arrest, a finding that has previously been reported in other cell types [30], and significantly reduced tumor mass in an in vivo mouse model [31]. Furthermore, S100A6 knockdown activated CXCL14-induced apoptosis in a renal cell carcinoma cell line [31]. In acute lymphoblastic leukemia cell lines, up-regulation of S100A6 was associated with reduced apoptosis due to interactions with the p53-caspase 8-caspase 3 pathway [32]. Pancreatic cancer cell lines showed significantly reduced cell proliferation and invasion after inhibition of S100A6 and different genes (e.g. human ovarian b-A inhibin, activin A and cytokine gro-b) that are known to be negative regulators of cell proliferation were shown to be up-regulated by S100A6 inhibition [33].

In cholangiocarcinoma, increased expression levels of S100A6 mRNA and protein were found in a small cohort of tissue specimens of intrahepatic CCA and it was suggested that S100A6 may help to differentiate between intrahepatic CCA and hepatocellular carcinoma (HCC) [34]. Moreover, other representatives of the S100 family such as S100A4 and S100P were shown to be overexpressed in CCA tumor samples and were associated with increased tumor invasiveness [35–37].

Although the cause and exact mechanism of S100A6 secretion into the bloodstream is not fully understood, different studies revealed elevated serum levels of S100A6 in NSCLC, gastric cancer and urothelial carcinoma [38–40] and suggested its role as a diagnostic and/or prognostic biomarker. As an example, S100A6 serum levels were significantly elevated in patients with gastric cancer and correlated with lymph node metastasis or TNM stage as well as overall survival [39].

Despite the emerging role of S100A6 in the pathophysiology of different (gastrointestinal) cancers, no data on the potential use of this ligand as a biomarker for cholangiocarcinoma are available. In the present study, we therefore analyzed serum levels of S100A6 in a cohort of 112 CCA patients that were admitted for surgery in curative intention at University Hospital RWTH Aachen, in order to determine the potential role of S100A6 as a diagnostic and prognostic biomarker for cholangiocarcinoma.

## Methods

### Study design and patient characteristics

This observational cohort study was designed to evaluate S100A6 as a diagnostic or prognostic serum marker for cholangiocarcinoma. Patients were enrolled from University Hospital RWTH Aachen and were prospectively recruited between 2011 and 2015. A total of 112 patients that were admitted to surgery for cholangiocarcinoma (56 % male, 44 % female, median age 68 years, range 37–84 years; see Tables 1 and 2) were included into this study. Serum samples were collected before any treatment prior to surgery and 6–7 days after tumor resection. As a control population, we analyzed 42 healthy, cancer-free blood donors with normal values for blood counts, C-reactive protein and liver enzymes. Moreover S100A6 concentrations were analyzed in 40 patients with histological confirmed HCC (Additional file 1: Table S1). The study protocol was approved by the local ethics committee and conducted in accordance with the ethical standards laid down in the Declaration of Helsinki (ethics committee of the University Hospital Aachen, RWTH University, Aachen, Germany). Written informed consent was obtained from each patient.

**Table 1:** Characteristics of CCA study population

Patients	112
Gender [%]	
Male–female	56–44
Age [median and range]	68 [37–84]
BMI [median and range]	26.5 [19.2–46.4]
Anatomic location of CCA [%]	
Intrahepatic	32
Klatskin	40
Distal	18
Gallbladder	10
Staging [%]	
T1–T2–T3–T4	4–41–32–23
N0–N1	39–61
M0–M1	75–25
G2–G3	58–42
R0–R1	57–43
UICC [%]	
I–II–III–IV	8–31–26–35
ECOG [%]	
ECOG 0	54
ECOG 1	36
ECOG 2	10
Fatigue [%]	
No	65
Low	22
Medium	10
High	3
Pain scale [%]	
0	83
1–3	10
4–6	7

**Table 2:** Levels of S100A6 and variant laboratory markers

	Median [range]
S100A6 [pg/ml]	
Healthy	2468.4 [0–10,841.5]
CCA patients pre-op	2085.8 [2–13,903.3]
CCA patients post-op	2197.7 [0–6817.8]
WBC [cells/µl]	8.5 [2.9–21.6]
CRP [mg/l]	18.8 [<5–230.0]
AST [U/l]	50 [18–1587]
ALT [U/l]	48 [10–1097]
GGT [U/l]	350 [36–2015]
ALP [U/l]	232 [53–1055]
Bilirubin [mg/dl]	1.1 [0.2–21.5]
Creatinine [mg/dl]	0.8 [0.5–1.6]
CEA [µg/l]	3.1 [0.7–110.4]
CA 19-9 [kU/l]	81 [0–18,854]

*WBC* white blood cell count; *CRP* C-reactive protein; *AST* aspartate transaminase; *ALT* alanine transaminase; *GGT* γ-Glutamyl transpeptidase; *ALP* alkaline phosphatase; *CEA* carcinoembryonic antigen; *CA 19-9* carbohydrate-Antigen 19-9

### Determination of serum S100A6

S100A6 serum concentrations were analyzed using a commercial enzyme-linked immunosorbent assay (ELISA) according to the manufacturer’s instructions (SEB769Hu by Cloud-Clone Corp., Houston, TX, USA). Evaluation of the ELISA absorbance values and calculation of the serum concentration was performed using a 4 Parameter Logistic (4PL) nonlinear regression model.

### Semi-quantitative reverse transcriptase PCR (qPCR)

RNA isolation from tissue samples, cDNA synthesis and qPCR was performed as recently described in detail [41, 42]. The following primers for S100A6 were used: 1. For human S100A6: 5′-TCTTCCACAAGTACTCCGGC-3′, 2. Rev human S100A6: 5′-TCCGGTCCAAGT-CTTCCATC-3′, 3. For murine S100A6: 5′-ACTCTGGCAAGGAAGGTGAC-3′, 4. Rev murine S100A6: 5′-GGCGACATACTCCTGGAAGT-3′. All qPCR reactions were performed in duplicates. Data were generated and analyzed using the SDS 2.3 and RQ manager 1.2 software packages (Applied Biosystems).

### CCA mouse model

For evaluation of murine S100A6 mRNA expression levels, tissue samples (tumor tissue, tumor microenvironment, healthy liver tissue from tumor-bearing mice and healthy liver tissue from untreated mice) from a recently published CCA mouse model were used [43]. In brief, the left liver lobe of six to eight-weeks-old p53^fl/fl^ mice (strain: C57BL/6) was electroporated with Sleeping Beauty-based oncogenic transposon plasmids. The combination of a targeted p53-knockout in hepatocytes and KRas-activation led to formation of a single intrahepatic cholangiocarcinoma within 3 weeks.

### Statistical analysis

Statistical analyses have been performed as recently described in detail [44]. In brief, data are given as median and range to reflect the skewed distribution of analysis on human samples. The Mann–Whitney-U-test and for multiple comparisons the Kruskal–Wallis-ANOVA was used. Box plot graphics display a statistical summary of the median, quartiles, ranges and extreme values. Correlations analyses were performed by using the Spearman correlation tests. The prognostic value of the variables was tested by univariate and multivariate analysis in the Cox regression model. Kaplan–Meier curves were plotted to display the impact on survival. ROC curves were generated by plotting sensitivity against one-specificity. All statistical analyses were performed with SPSS (SPSS, Chicago, IL, USA) and GraphPad Prism 5.0 (GraphPad Software Inc., La Jolla, CA, USA) [45].

## Results

### S100A6 expression is up-regulated in tumor tissue of human and murine CCA

**Fig. 1:**
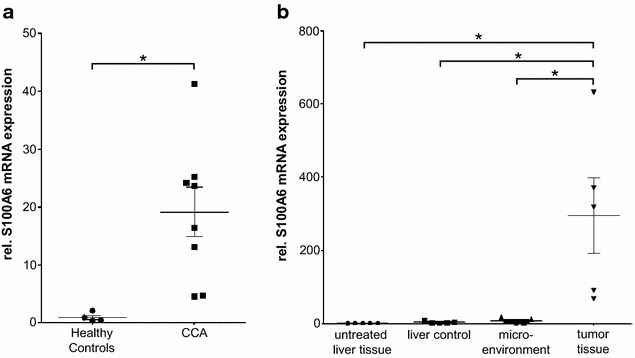
S100A6 mRNA levels are up-regulated in human and murine CCA tissue samples. **a** S100A6 mRNA expression levels, measured by qPCR analysis, are significantly up-regulated in tumor samples of CCA patients (n = 8) compared to healthy controls (n = 4). **b** In murine CCA tumor tissue (n = 5), S100A6 is overexpressed when compared to liver tissue from untreated mice, liver control tissue from tumor-bearing mice and the microenvironment of the tumor

To confirm the recently suggested overexpression of S100A6 mRNA in tumor samples of patients with intrahepatic CCA, we analyzed tumor S100A6 mRNA levels in a cohort of patients with histologically confirmed CCA (n = 8) by using qPCR and compared them to healthy controls (n = 4). In accordance to the existing literature, this analysis revealed significantly elevated S100A6 expression levels in tissue samples of CCA patients (Fig. 1a). To further confirm this finding, we next examined S100A6 mRNA expression levels in a recently published CCA mouse model. Similar to the human tumor expression patterns, this analysis also showed a significantly up-regulated S100A6 expression in CCA tumor tissue compared to normal liver tissue of untreated or tumor-bearing mice as well as the tumor microenvironment (Fig. 1b).

### Serum S100A6 levels in patients with CCA

**Fig. 2:**
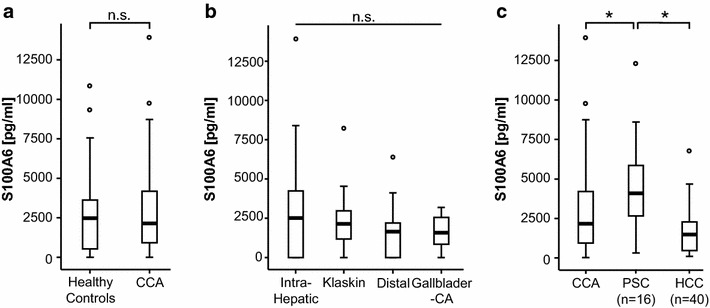
Analysis of S100A6 levels is not suitable for diagnosis of CCA. **a** S100A6 levels measured by commercially available ELISA at the time point of admission to our hospital were unaltered in patients with CCA (n = 112) compared to healthy controls (n = 42). **b** No significant alterations were observed between intra-hepatic CCA, Klatskin tumors, distal CCA and gallbladder cancer. **c** Patients with a CCA due to a PSC displayed significantly higher serum concentrations than other patients. No alterations in S100A6 serum levels were found for HCC patients

Based on the up-regulated expression levels of S100A6 in human and murine CCA tissue samples and the available data suggesting a role for S100A6 in the pathophysiology of various gastrointestinal cancers, including the potential use of serum S100A6 levels as diagnostic biomarker, we next analyzed serum levels of S100A6 in a large and well characterized cohort of 112 patients with CCA and compared them to healthy controls (patients characteristics are given in Table 1). Unexpectedly, this analysis revealed no difference in serum concentrations between CCA-patients and healthy controls (Fig. 2a). To uncover differences in S100A6 serum concentrations that might be restricted to specific subgroups CCA patients, we next analyzed S100A6 levels in patients with different types of CCA. However, no significant alterations were observed between intra-hepatic CCA, Klatskin tumors, extra-hepatic CCA and gallbladder cancer (Fig. 2b). Interestingly, patients with a CCA due to a primary sclerosing cholangitis displayed significantly higher serum concentrations than all other patients, which is in line with previous reports demonstrating higher levels of the S100 protein family members in inflammatory and immunological diseases (Fig. 2c) [46–48]. Finally, serum concentration levels of S100A6 were also not significantly altered in patients with hepatocellular carcinoma (Fig. 2c).

**Fig. 3:**
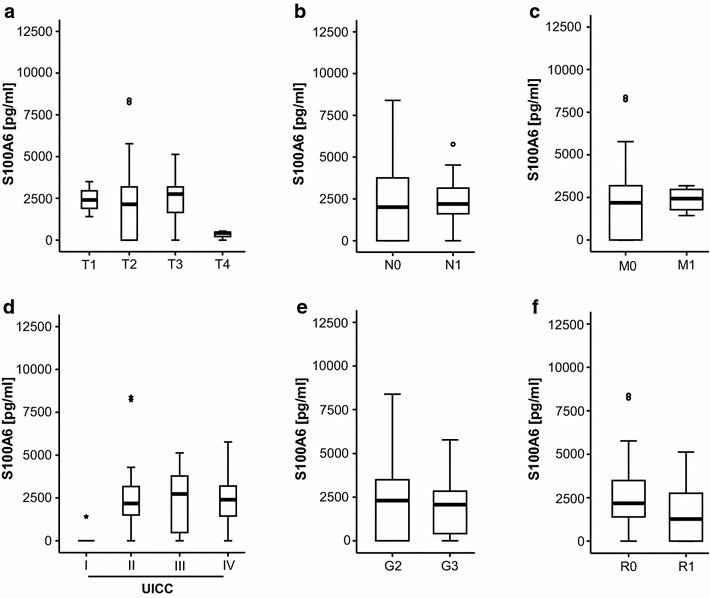
S100A6 levels do not correlate with tumor stage, tumor differentiation or resection status. **a**–**e** S100A6 levels at time point of admission to our hospital were unaltered in patients with different T-status, nodal positive vs. negative disease, metastasized vs. non-metastasized disease, different UICC-stadiums and different tumor grading. **f** S100A6 levels at time point of admission to our hospital were unaltered in patients with R0 vs. R1 resection

We next examined S100A6 levels in different stages of disease (TNM and UICC classification, nodal negative vs. positive, non-metastasized vs. metastasized, well- vs. undifferentiated). However, this analysis revealed no significant differences between the different subgroups of patients (Fig. 3a–e). Finally, pre-operative serum levels of S100A6 were not altered in patients with incomplete tumor resection (R1), compared to patients with complete resection (R0) and did not correlate with clinical symptoms of CCA such as fatigue, pain or impaired ECOG performance status (Fig. 3f and Additional file 2: Figure S1). To identify factors potentially regulating serum S100A6 serum concentrations, we next analyzed correlations between S100A6 and routinely used laboratory markers in patients with cancer, but also this analysis revealed no clear correlation (Table 3). In summary, our data suggest that in contrary to other gastrointestinal cancers, measurement of S100A6 is unsuitable as a diagnostic serum biomarker in patients with cholangiocarcinoma.

**Table 3:** Correlation analysis between S100A6 and variant laboratory markers (R, Spearman coefficient; p, *p* value)

	S100A6 pre-op		S100A6 post-op	
	R	p	R	p
S100A6 pre-op	–	–	0.315	0.057
S100A6 post-op	0.315	0.057	–	–
WBC	0.132	0.241	0.144	0.381
CRP	0.161	0.162	0.285	0.083
AST	−0.058	0.608	0.016	0.924
ALT	−0.060	0.656	0.007	0.974
Bili	−0.160	0.157	0.162	0.325
GGT	−0.119	0.293	0.069	0.677
AP	−0.077	0.499	0.063	0.705
Creatinine	0.137	0.227	0.434	0.006
CEA	0.232	0.201	0.104	0.734
CA 19-9	0.025	0.889	0.202	0.488
LDH	0.126	0.509	0.199	0.126

### Postoperative serum levels of S100A6 do not reflect tumor characteristics

**Fig. 4:**
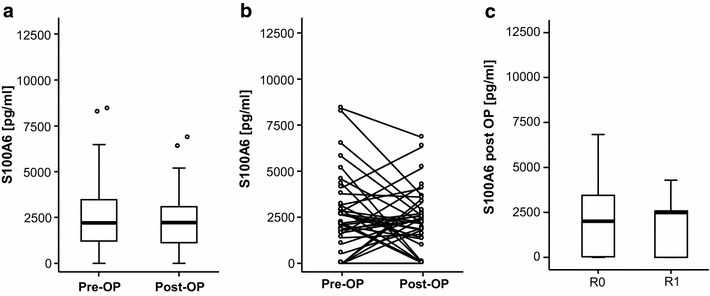
Postoperative serum levels of S100A6 do not reflect tumor characteristics. **a** and **b** Serum levels of S100A6 were not significantly altered after tumor resection when compared to levels at time point of admission to our hospital. **c** Postoperative S100A6 concentrations were similar in patients with complete vs. incomplete tumor resection

For 33 patients blood serum concentrations of S100A6 were available postoperatively. We analyzed if, in contrary to initial S100A6 levels, postoperative S100A6 concentrations might have a better value for determination of tumor characteristics or success of surgery. Postoperative S100A6 concentrations were similar in patients with complete vs. incomplete tumor resection and in patients with well-differentiated vs. undifferentiated tumors. Furthermore, the tumor stages had no influence on S100A6 serum levels (Fig. 4 and Additional file 3: Figure S2).

### Serum S100A6 levels as prognostic biomarker for CCA

**Fig. 5:**
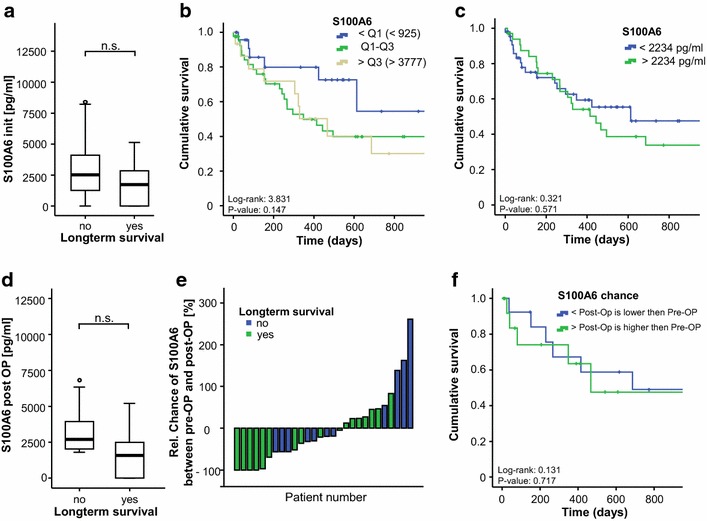
Serum S100A6 levels as prognostic biomarker for CCA. **a** Patients that succumbed to death showed a trend towards higher levels of S100A6 at admission to our hospital when compared to survivors (not significant). **b**, **c** Cox regression- and Kaplan–Meyer curve analyses revealed that those patients that displayed high S100A6 levels (e.g. S100A6 levels within the upper quartile of the CCA cohort) had a trend towards an impaired prognosis. **d** Postoperative S100A6 levels also depicted a trend towards a better survival in patients with low S100A6 concentrations. **e**, **f** Kinetics of S100A6 serum concentrations before/after surgery did not indicate the patients’ prognosis

To identify a potential association between initial S100A6 serum concentrations and patients' outcome we next compared the preoperative concentrations in patients that succumbed to death during the follow-up period with that of survivors. We observed a trend towards higher pre-surgery levels of S100A6 in patients that succumbed to death compared to the other patients (Fig. 5a). To analyze the prognostic accuracy of S100A6 serum concentrations we further performed Cox regression- and Kaplan–Meyer curve analyses. These analyses confirmed the observation that those patients that displayed high S100A6 levels (e.g. S100A6 levels within the upper quartile of the CCA cohort) had a trend towards an impaired prognosis (Fig. 5b, c). Further analyses revealed that the prognostic value of S100A6 was slightly inferior to INR but superior to CRP, creatinine and patients’ age according to ROC curve analysis (Additional file 4: Figure S3). Moreover, we applied the Youden-index method to define the best cut-off value to discriminate survivors from non-survivors. This analysis revealed that patients with S100A6 levels of lower than 2234 pg/ml had a slightly (yet not significantly) better prognosis compared to the other patients (Fig. 5c). Notably, when postoperative S100A6 levels were analyzed with respect to patients’ survival we confirmed the trend towards a better survival in patients with low S100A6 concentrations (Fig. 5d). We next examined whether alterations of S100A6 serum concentrations before and after surgery might indicate patients’ prognosis. However, the differences between S100A6 levels at admission to the hospital and after surgery did not reflect patients’ prognosis (Fig. 5e, f).

### Comparison of serum S100A6 concentrations and CEA or CA19-9 serum levels

In clinical routine, measurements of CEA and CA19-9 are frequently performed in patients with CCA. We therefore compared the diagnostic and prognostic power of S100A6 measurements to those of CEA and CA19-9 in our cohort of patients with CCA. Similar to S100A6, both CEA and CA19-9 were not significantly elevated in patients with CCA. Concentrations of these markers did not differ in patients that succumbed to death and survivors (Additional file 5: Figure S4a and b). Notably, ROC curve analysis revealed that neither CEA nor CA19-9 were significantly superior to S100A6 measurements in prediction of patients' outcome (Additional file 5: Figure S4c).

## Discussion

Although the incidence of CCA is rising worldwide, knowledge on biomarkers for this heterogeneous disease allowing to non-invasively establish the diagnosis (e.g., in high-risk patients with cholestatic diseases) or to predict prognosis is scarce [49]. In the present study, we demonstrate that S100A6 expression levels are significantly up-regulated in CCA tumor tissue but measurements of circulating S100A6 are not suitable for the diagnosis of CCA. Nevertheless, S100A6 serum levels might be indicative for the prognosis of patients with CCA. Of note, within our cohort of patients, measurements of S100A6 demonstrated a similar prognostic power than that of clinically established biomarkers such as CEA or CA19-9.

In humans, the S100 protein family consists of about 20 members that are characterized by a similar structure and all act as modulators of cellular responses towards injury and stress [50]. Alterations in the expression of S100 family members represent a common feature of several cancers including gastrointestinal malignancies such as gastric cancer, pancreatic cancer and colorectal adenocarcinoma [25, 26, 39, 51]. While the expression of different S100 members is commonly up-regulated in malignant tumors, distinct and in part contradictory expression profiles were observed in many cancers and may be attributable to cancer subtype, disease stage, cellular distribution, or issues associated with S100 protein and/or mRNA detection. As an example, S100A11 expression is increased in NSCLC, but is decreased in small-cell lung cancer [52]. Similarly, we did not detect a significant alteration in S100A6 serum levels in cholangiocarcinoma, while an up-regulation was described in other gastrointestinal cancers [25, 39]. In this context, it was recently suggested that differences in S100A6 expression might help to distinguish between cholangiocarcinoma and hepatocellular cancer as S100A6 expression levels were described to be up-regulated in CCA tumor tissue but normal in HCC tissue samples [34]. In accordance with this finding, we showed that S100A6 is significantly overexpressed in human and murine CCA tissue samples, corroborating a pathophysiological role of S100A6 in cholangiocarcinoma. However, at least in our cohort of patients, these data were not reflected by concordant alterations in blood or serum levels (Fig. 2c), highlighting the complexity in the regulation of S100 family members in cancers.

We found slight alterations in S100A6 levels in patients that succumbed to death compared to survivors. Moreover, patients with S100A6 serum levels of less than 2234 ng/ml displayed a trend towards a better survival in Kaplan–Meier curve analysis, highlighting that S100A6 serum levels might reflect prognosis-relevant processes in patients with CCA. This justifies to further evaluate S100A6 in larger prospective trials, possibly alongside other promising biomarker candidates in order to develop new multi-parametric prognostic scores. Similar observations were made for S100A2 as a negative prognostic biomarker in pancreatic cancer [53]. In line, S100 family members, including S100A6, have been demonstrated to regulate critical processes such as tumor growth, metastasis, angiogenesis and immune evasion [54–57].

Recently, serum levels of S100A2 and S100A6 were found to be higher in patients with NSCLC compared to controls [38]. Moreover, also in patients with gastric cancer, serum S100A6 levels were elevated and significantly correlated with lymph node metastasis, TNM stage, perineural invasion and vascular invasion, supporting that S100A6 might represent a novel marker indicating patients prognosis independently of the examined tumor entity [39]. We therefore hypothesized that also differences between pre- and postoperative levels in S100A6 levels might be of prognostic relevance. However, no significant difference became apparent when we compared pre- and postoperative concentration of S100A6. The results were not predictive for tumor or surgery specific factors such as complete tumor resection or tumor grading, which might have been expected as members of the S100 protein family have previously been shown to serve as a biomarker for the evaluation of response to surgical treatment and prediction of early relapse and survival in melanoma [58].

Recent data suggested a prognostic function of CA19-9 but not CEA in patients with CCA [59, 60]. In our cohort of patients no significant differences in survival for patients with elevated CEA or CA19-9 serum concentrations compared to those with lower levels became apparent. Importantly, both of these routinely used markers displayed a lower prognostic accuracy than S100A6, highlighting that new and innovative biomarkers might be superior in this setting than currently used markers. Beside S100A6 a prognostic function has also been described for cytokeratin-19 fragments (CYFRA 21-1), IL-6 and microRNA-21, but the available data suggest that panels of more than one biomarker are needed to reliably predict prognosis in patients with CCA [61–63].

Guiding early therapeutic decisions represents an important challenge in the treatment of patients with cholangiocarcinoma. In light of our findings that S100A6 concentrations might be indicative for an impaired prognosis, our results indicate that measurement of circulating S100A6 levels could be considered as a novel element in the diagnostic algorithm of patients with cholangiocarcinoma. Of note, although in our patients the prognostic value of S100A6 serum levels for overall survival was limited, it was similar to that of established prognostic markers such as CA19-9. As almost all of our patients underwent surgery for CCC and only few patients displayed a metastasized stage of disease already at the time-point of diagnosis, we cannot fully rule out that the findings of our study are only valid for this subgroup of patients and that S100A6 levels might be different in patients with metastases. Thus, future studies combining prospective clinical trials and experimental animal models are needed to further establish S100A6 measurements in the diagnosis of cholangiocarcinoma and to define the exact role of S100 family members in the pathophysiology of this devastating disease.

## Conclusion

Our study, including 112 patients with histologically confirmed CCA, demonstrates that despite a significantly up-regulated expression of S100A6 in human and murine CCA tumor samples, S100A6 serum levels are not suitable as a biomarker for the diagnosis of CCA, which is in contrast to previous findings, suggesting a role of serum S100A6 as a biomarker in other gastrointestinal malignancies. CCA-patients with elevated S100A6 levels show a trend toward an impaired prognosis when compared to patients with lower S100A6 levels. This finding supports further evaluation of S100A6 as a prognostic biomarker in CCA.

## Additional files

**Additional file 1: Table 1.** Characteristics of HCC study population.

**Additional file 2: Fig. S1.** Serum measurements of S100A6 do not reflect clinical features of CCA such as fatigue (A), pain (B) or an impaired ECOG performance status (C).

**Additional file 3: Fig. S2.** Postoperative S100A6 concentrations were similar in patients with different T stages (A), nodal positive vs. negative disease (B), UICC-stadiums (C), well-differentiated vs. undifferentiated tumors (D).

**Additional file 4: Fig. S3.** ROC curve analysis showed that the prognostic value of S100A6 was slightly inferior to INR but superior to CRP, creatinine and patients’ age.

**Additional file 5: Fig. S4.** (A and B) Concentrations of CEA and CA19-9 did not differ in patients that succumbed to death and survivors. ROC curve analysis revealed that neither CEA nor CA19-9 were significantly superior to S100A6 measurements in prediction of patients’ outcome.

## Abbreviations

S100A6: S100 calcium-binding protein A6
CCA: cholangiocarcinoma
PSC: primary sclerosing cholangitis
HCC: hepatocellular carcinoma
UICC: Union for International Cancer Control
ECOG: Eastern Cooperative Oncology Group
INR: International Normalized Ratio
NSCLC: non-small-cell lung carcinoma
CYFRA: cytokeratin-19 fragments
WBC: white blood cell count
CRP: C-reactive protein
AST: aspartate transaminase
ALT: alanine transaminase
GGT: γ-Glutamyl transpeptidase
ALP: alkaline phosphatase
CEA: carcinoembryonic antigen
CA 19-9: carbohydrate-Antigen 19-9
BMI: body mass index
qPCR: semi-quantitative reverse transcriptase PCR
