# Supplementary material for: Serum levels of S100A6 are unaltered in patients with resectable cholangiocarcinoma
Source: Clin Transl Med. 2016 Sep 27;5:39. doi: 10.1186/s40169-016-0120-7 (PMC5052241; doi:10.1186/s40169-016-0120-7)
Supplement: Supplementary file 1 — Additional file 1: Table 1. Characteristics of HCC study population. [file 40169_2016_120_MOESM1_ESM.docx]

**Supplementary Table 1**. Characteristics of HCC study population

|  |  |
| --- | --- |
| Patients | 40 |
| Gender [%]:  male-female | 76-24 |
| Age [median and range] | 63 [32-82] |
| BMI [median and range] | 26.3 [17.8-38.2] |
| ECOG [%]  ECOG 0  ECOG 1  ECOG 2 | 63  33  4 |
| Fatigue [%]  No  Low  Medium  High | 48  25  20  7 |
| Pain scale [%]  0  1-3  4-6 | 90  10  0 |
|  |  |
