# Supplementary material for: Serum levels of S100A6 are unaltered in patients with resectable cholangiocarcinoma
Source: Clin Transl Med. 2016 Sep 27;5:39. doi: 10.1186/s40169-016-0120-7 (PMC5052241; doi:10.1186/s40169-016-0120-7)
Supplement: Supplementary file 2 — Additional file 2: Fig. S1. Serum measurements of S100A6 do not reflect clinical features of CCA such as fatigue (A), pain (B) or an impaired ECOG performance status (C). [file 40169_2016_120_MOESM2_ESM.ppt]

## Slide 1
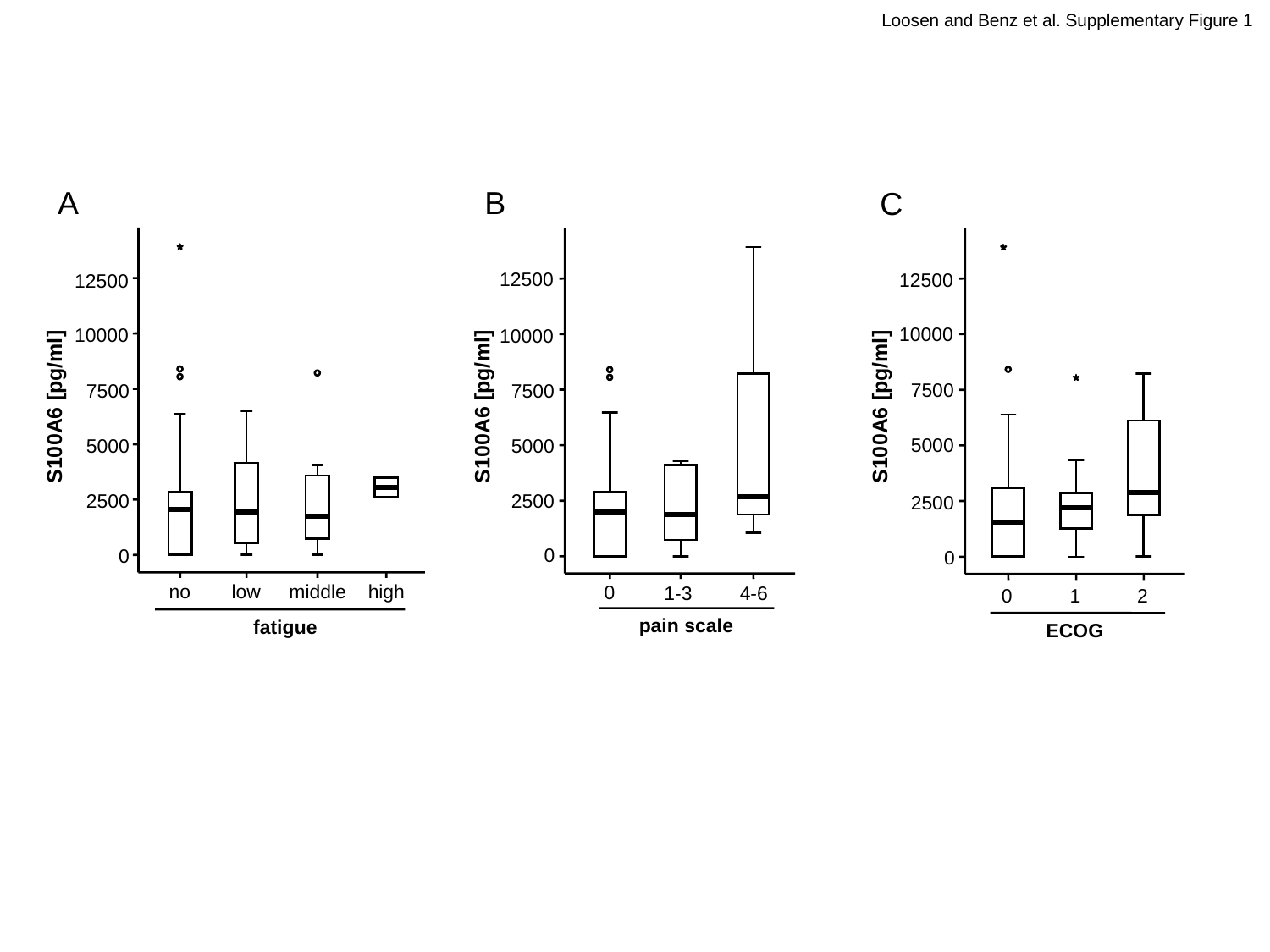

Loosen and Benz et al. Supplementary Figure 1
A
B
C
12500
10000
S100A6 [pg/ml]
7500
5000
2500
0
no
low
middle
high
fatigue
12500
10000
S100A6 [pg/ml]
7500
5000
2500
0
0
1-3
4-6
pain scale
12500
10000
S100A6 [pg/ml]
7500
5000
2500
0
0
1
2
ECOG
