# Supplementary material for: Serum levels of S100A6 are unaltered in patients with resectable cholangiocarcinoma
Source: Clin Transl Med. 2016 Sep 27;5:39. doi: 10.1186/s40169-016-0120-7 (PMC5052241; doi:10.1186/s40169-016-0120-7)
Supplement: Supplementary file 3 — Additional file 3: Fig. S2. Postoperative S100A6 concentrations were similar in patients with different T stages (A), nodal positive vs. negative disease (B), UICC-stadiums (C), well-differentiated vs. undifferentiated tumors (D). [file 40169_2016_120_MOESM3_ESM.ppt]

## Slide 1
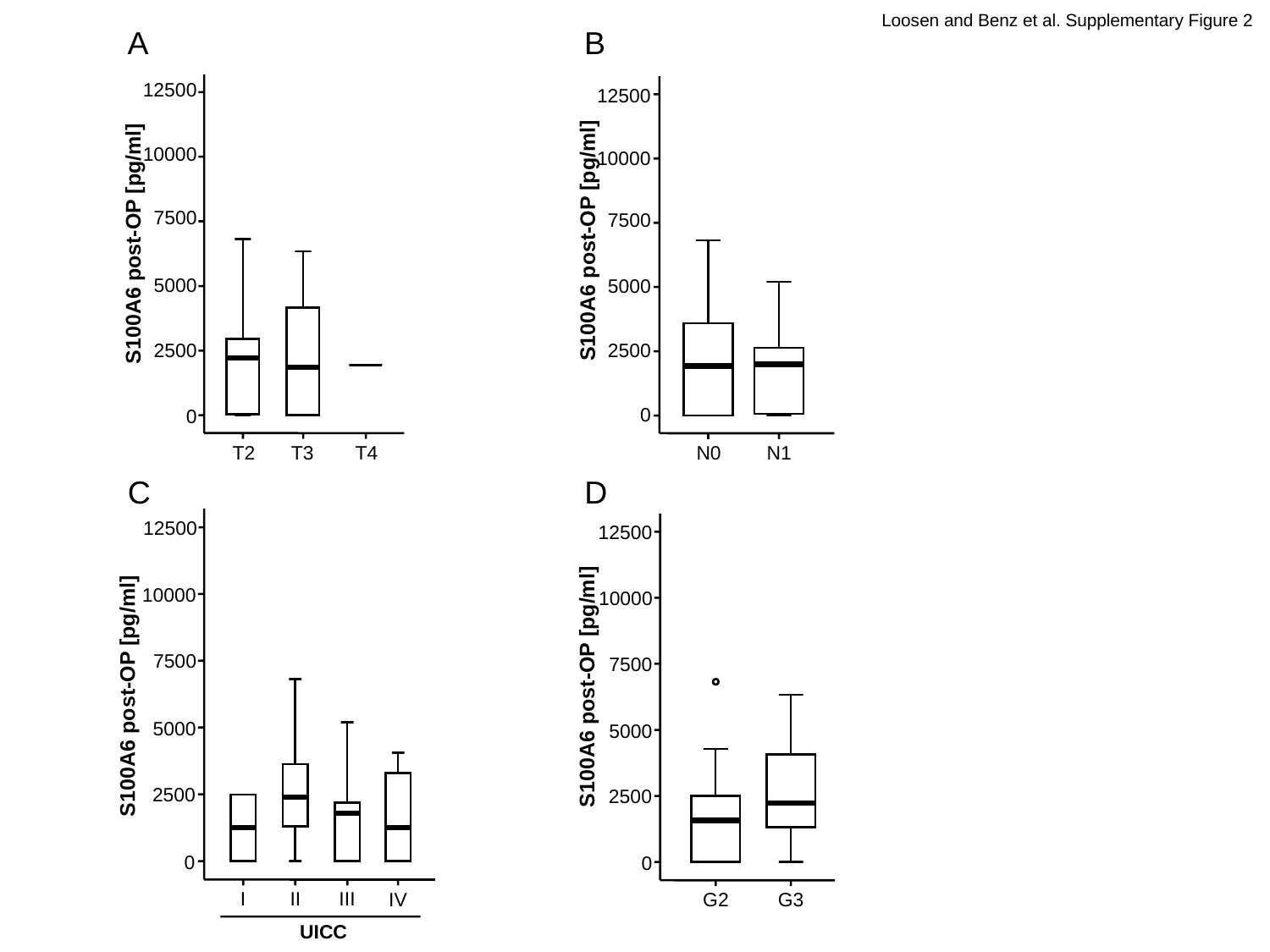

Loosen and Benz et al. Supplementary Figure 2
A
B
12500
10000
7500
S100A6 post-OP [pg/ml]
5000
2500
0
T2
T3
T4
12500
10000
7500
S100A6 post-OP [pg/ml]
5000
2500
0
N0
N1
C
D
12500
10000
7500
S100A6 post-OP [pg/ml]
5000
2500
0
I
II
III
IV
12500
10000
7500
S100A6 post-OP [pg/ml]
5000
2500
0
G2
G3
UICC
