# Supplementary material for: Serum levels of S100A6 are unaltered in patients with resectable cholangiocarcinoma
Source: Clin Transl Med. 2016 Sep 27;5:39. doi: 10.1186/s40169-016-0120-7 (PMC5052241; doi:10.1186/s40169-016-0120-7)
Supplement: Supplementary file 4 — Additional file 4: Fig. S3. ROC curve analysis showed that the prognostic value of S100A6 was slightly inferior to INR but superior to CRP, creatinine and patients’ age. [file 40169_2016_120_MOESM4_ESM.ppt]

## Slide 1
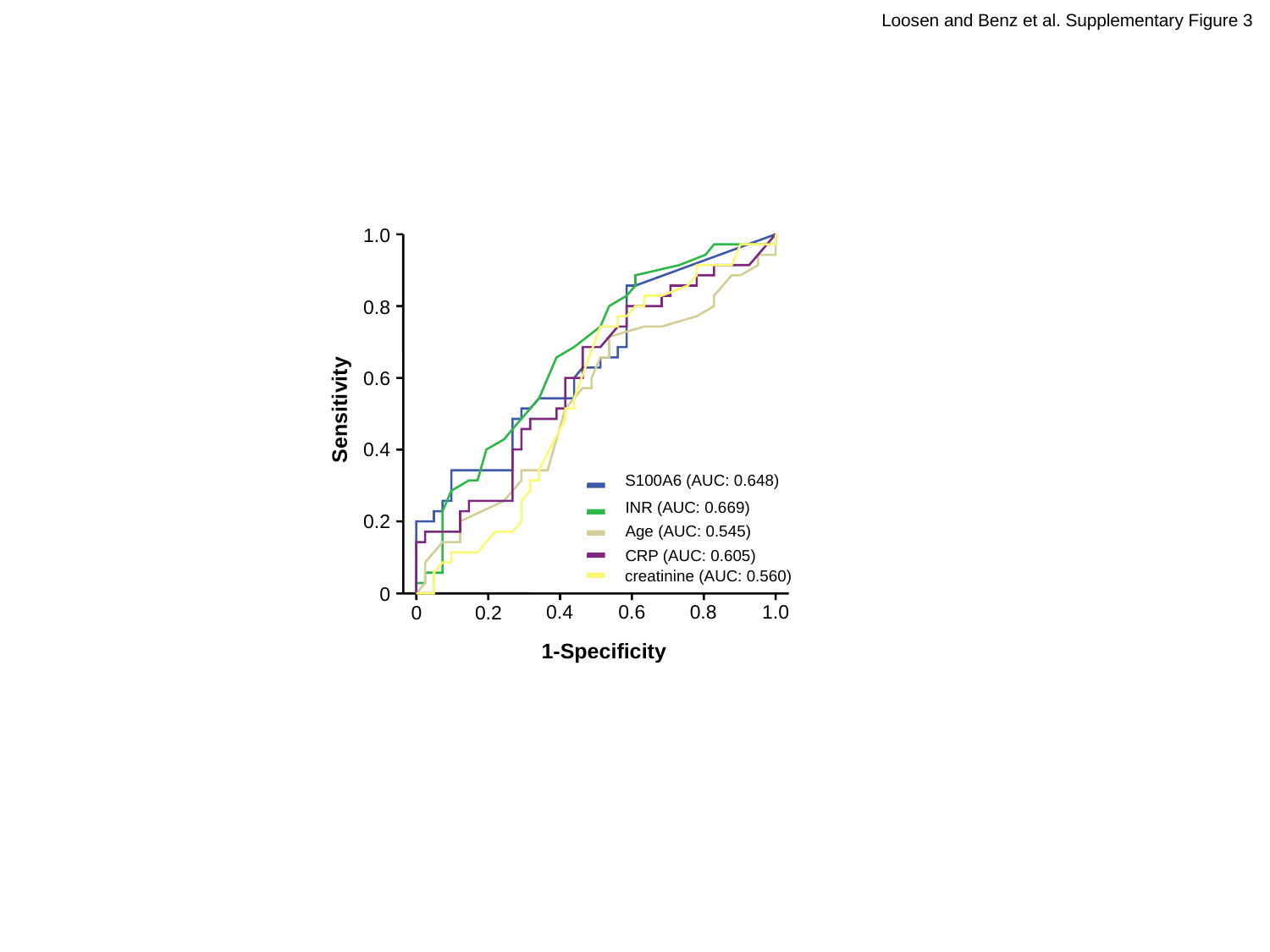

Loosen and Benz et al. Supplementary Figure 3
1.0
0.8
Sensitivity
0.6
0.4
S100A6 (AUC: 0.648)
INR (AUC: 0.669)
0.2
Age (AUC: 0.545)
CRP (AUC: 0.605)
creatinine (AUC: 0.560)
0
1-Specificity
0.4
0.6
0.8
1.0
0.2
0
