# Supplementary material for: Serum levels of S100A6 are unaltered in patients with resectable cholangiocarcinoma
Source: Clin Transl Med. 2016 Sep 27;5:39. doi: 10.1186/s40169-016-0120-7 (PMC5052241; doi:10.1186/s40169-016-0120-7)
Supplement: Supplementary file 5 — Additional file 5: Fig. S4. (A and B) Concentrations of CEA and CA19-9 did not differ in patients that succumbed to death and survivors. ROC curve analysis revealed that neither CEA nor CA19-9 were significantly superior to S100A6 measurements in prediction of patients’ outcome. [file 40169_2016_120_MOESM5_ESM.ppt]

## Slide 1
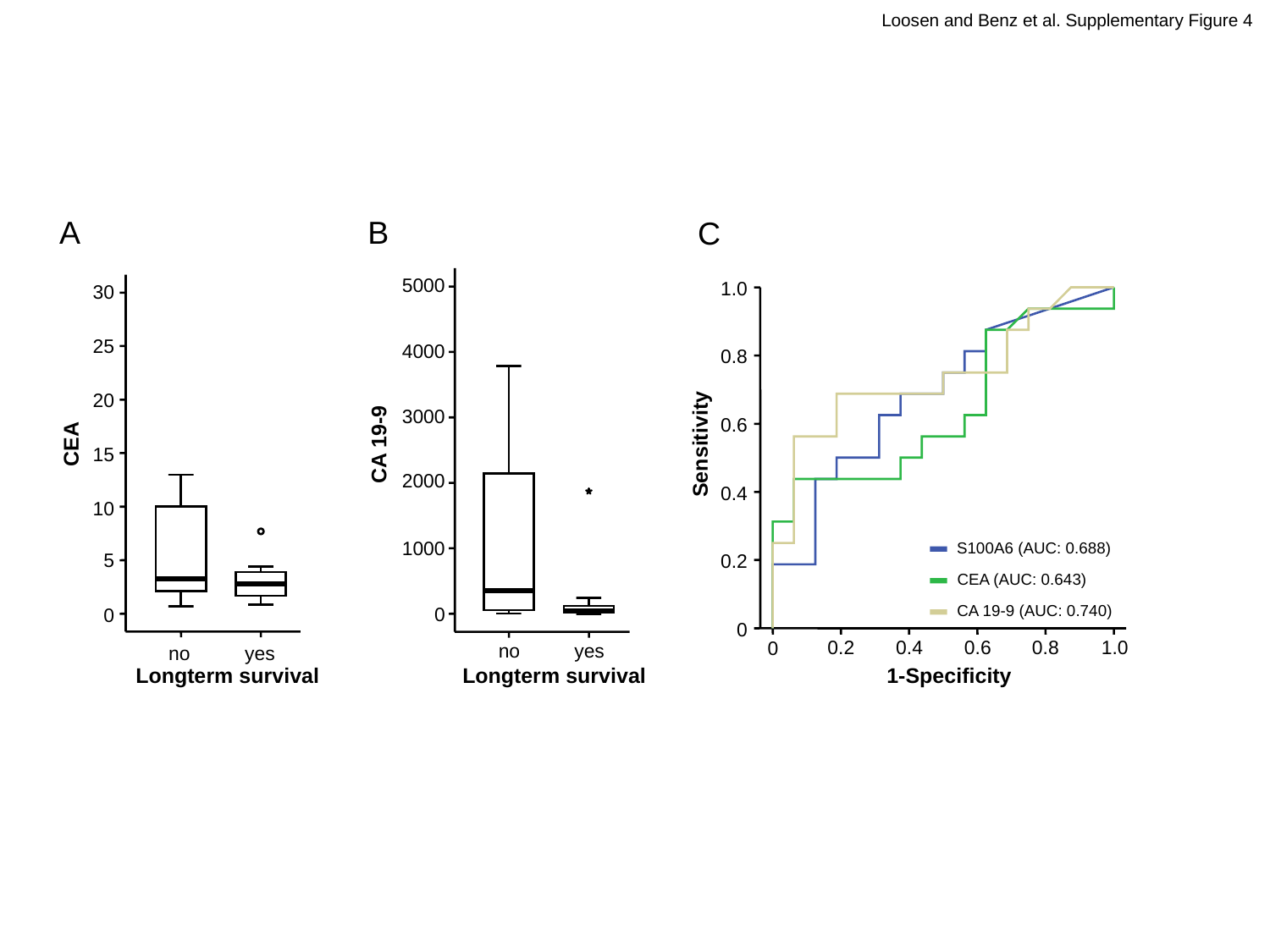

Loosen and Benz et al. Supplementary Figure 4
A
B
C
5000
1.0
30
25
4000
0.8
20
Sensitivity
3000
CA 19-9
0.6
CEA
15
2000
0.4
10
1000
S100A6 (AUC: 0.688)
5
0.2
CEA (AUC: 0.643)
Longterm survival
Longterm survival
CA 19-9 (AUC: 0.740)
0
0
1-Specificity
0
0.2
0.4
0.6
0.8
1.0
0
no
yes
no
yes
